# Genomic and Gut Microbiome Evaluations of Growth and Feed Efficiency Traits in Broilers

**DOI:** 10.3390/ani14243615

**Published:** 2024-12-15

**Authors:** Xia Xiong, Chunlin Yu, Mohan Qiu, Zengrong Zhang, Chenming Hu, Shiliang Zhu, Li Yang, Han Peng, Xiaoyan Song, Jialei Chen, Bo Xia, Jiangxian Wang, Yi Qing, Chaowu Yang

**Affiliations:** 1 Animal Breeding and Genetics Key Laboratory of Sichuan Province, Sichuan Animal Science Academy, Chengdu 610066, China; xiongxia20120904@163.com (X.X.); yuchunlin1984@sina.com (C.Y.); mohan.qiu@163.com (M.Q.); zhangzengrong2004@163.com (Z.Z.); huchenming@126.com (C.H.); zhushiliang1994@163.com (S.Z.); yangli_sasa@163.com (L.Y.); penghan0706@163.com (H.P.); babalasxy@163.com (X.S.); qiaoqiaowo110@163.com (J.C.); allanbobo777@163.com (B.X.); wangjiangxian@scsaas.cn (J.W.); 2 Chengdu Livestock and Poultry Genetic Resources Protection Center, Chengdu 610081, China

**Keywords:** average daily gain, chicken, feed conversion ratio, genomic selection, residual feed intake

## Abstract

In this study, we combined genomic and gut microbiome data to evaluate 13 economically important growth and feed efficiency traits in 407 Dahen broilers, including body weight (BW) at four, six, nine, and ten weeks of age (BW4, BW6, BW9, and BW10), as well as the average daily gain (ADG6, ADG9, and ADG10), feed conversion ratio (FCR6, FCR9, and FCR10), and residual feed intake (RFI6, RFI9, and RFI10) for the three growing ages. The highest ADG and lowest FCR were observed at nine and six weeks of age, respectively. We obtained 47,872 high-quality genomic single-nucleotide polymorphisms (SNPs) by sequencing the genomes and 702 amplicon sequence variants (ASVs) of the gut microbiome by sequencing the 16S rRNA gene, both of which were used for analyses of linear mixed models. The heritability estimates (± standard error, SE) ranged from 0.103 ± 0.072 to 0.156 ± 0.079 for BW, 0.154 ± 0.074 to 0.276 ± 0.079 for the ADG, 0.311 ± 0.076 to 0.454 ± 0.076 for the FCR, and 0.413 ± 0.077 to 0.609 ± 0.076 for the RFI traits. We consistently observed moderate and low negative genetic correlations between the BW traits and the FCR and RFI traits (r = −0.562 to −0.038), whereas strong positive correlations were observed between the FCR and RFI traits (r = 0.564 to 0.979). For the FCR and RFI traits, strong positive correlations were found between the measures at the three ages. In contrast to the genomic contribution, we did not detect a gut microbial contribution to all of these traits, as the estimated microbiabilities did not confidently deviate from zero. We systematically evaluated the contributions of host genetics and gut microbes to several growth and feed efficiency traits in Dahen broilers, and the results show that only the host genetics had significant effects on the phenotypic variations in a flock. The parameters obtained in this study, based on the combined use of genomic and gut microbiota data, may facilitate the implementation of efficient breeding schemes in Dahen broilers.

## Simple Summary

This study was conducted to investigate feed efficiency in chickens. Growth and feed efficiency traits were evaluated in Dahen chickens based on genomic and gut microbiome data. The results demonstrate that host genetics exert a significant influence on phenotypic variations in this population. The findings of this study serve to establish a foundation and provide a data reference point for the field of broiler breeding.

## 1. Introduction

Dahen broilers are commercial hybrids derived from three specialized lines that have been recently developed in Sichuan, China. The Dahen broiler has a typical yellow feather and red crown appearance, good meat quality, and excellent production performance, making it one of the main breeds in southwest China. At ten weeks of age, the commercial generation of Dahen broilers produces an average body weight (BW) of 2668 g for males and 2269 g for females, and the feed conversion ratio (FCR) between 1 and 70 days of age is about 2.3. Compared to other commercial hybrids, such as the Green leg chicken and Lingnan yellow chicken, Dahen broilers have a relatively high adult weight. However, another obvious advantage of Dahen broilers is that they are well known for their excellent meat quality, as it is rich in nutrition, has a delicious taste and high safety standards, and aligns with sustainable farming practices [1]. In addition to growth performance, continuous efforts have been dedicated to genetically improving the feed efficiency in Dahen broilers; this had not been routinely included as a direct selection trait in previous breeding programs. In fact, it is a highly time-consuming and expensive task to collect large-scale phenotypic records on feed efficiency, which prevents its direct selection when using traditional approaches [2].

Due to their considerable heritabilities, easy-to-measure phenotypes, and direct impact on meat production, BW traits measured at different ages have been the prime selection traits in recent decades [3,4,5]. In addition to the absolute growth rate, determining how to genetically improve the feed efficiency has also attracted increasing attention in recent years, especially in the context of addressing global food security and reducing carbon footprints [6]. Although the FCR in broilers at five weeks of age had decreased from 2.3 in 1985 to 1.5 in 2010 [7], this would have mostly indirectly resulted from intense selection for the growth rate. Sell-Kubiak et al. [8] provided a great review on the genetic aspects of feed efficiency traits in meat-type chicken, including the various measures used, genetic architecture, and selection strategies. Overall, it seems feasible to genetically improve feed efficiency as the evaluated measures, such as the FCR and residual feed intake (RFI), have moderate or high heritability estimates in different populations of broilers [9,10,11]. If the heritability is high, we could effectively select individuals directly based on the phenotypic values. Furthermore, candidate genes and quantitative trait loci associated with feed efficiency have been widely explored in broilers [12,13,14]; for example, a genomic region (Chr1: 91.27–92.43 Mb) was found to be significantly associated with the RFI, together with the potential candidate genes NSUN3 and EPHA6.

Over the past decade, genomic selection technologies have profoundly revolutionized livestock and poultry breeding programs [15]. One of the benefits of genomic selection is the potential application to hard-to-measure traits, such as feed efficiency, meat quality, and welfare, as individual phenotypes are not required in the selection population. Therefore, genomic selection has been proposed to improve feed efficiency in various livestock and poultry species, such as broilers [16,17], pigs [18], dairy cattle [19], and beef cattle [20]. Wolc et al. [21] specifically discussed the implementation of genomic selection in broilers and layers, as well as the distinctive features of the poultry industry. Besides host genetics, gut microbes have been increasingly found to positively influence feed efficiency and growth performance in broilers [22,23,24,25]. However, it is well known that the relative abundances of some gut microbes are heritable [26,27]. In this context, it can be expected that some of the host genetic effects on traits may be indirectly mediated by the gut microbial community, which hence leads to the requirement of a joint evaluation combining host genomic and gut microbiome data [23,28,29]. Therefore, in this study, we similarly evaluated genomic and gut microbial contributions to the growth and feed efficiency traits in Dahen broilers, aiming to determine the parameters necessary for implementing efficient selection based on genomic and gut microbiota data.

## 2. Materials and Methods

### 2.1. Animals and Phenotypes

This animal study was approved by the Animal Ethics Committee of Sichuan Animal Science Academy (202214865). Dahen broilers are commercial hybrids derived from three specialized lines that have been recently developed in Sichuan, China. At ten weeks of age, the commercial generation of Dahen broilers produces an average body weight (BW) of 2668 g for males and 2269 g for females, and the feed conversion ratio (FCR) between 1 and 70 days of age is about 2.3. A total of 460 males from the Dahen broilers S09 line were included in this study; they were hatched on the same day and provided by Sichuan Dahen Poultry Breeding Co., LTD. All the broilers were housed in floor pens with fresh wood sawdust before four weeks of age; the stocking density was about 15 per m^2^, with a temperature range of 16–27 °C and lighting controls set to 16 h of light followed by 8 h of darkness. Between four and ten weeks of age, all the broilers were individually housed and fed ad libitum with water and a commercial pellet diet (the feed ingredients and nutritional values are presented in Supplementary File S1: Table S1), and the weekly BW (g) and feed intake (FI, g) were individually recorded. In addition to the BW measured at four, six, nine, and ten weeks of age (BW4, BW6, BW9, and BW10), we further calculated the individual average daily gain (ADG, g/day), FCR (the ratio of feed intake to weight gain), and RFI (g/day) at three growth intervals: between four and six weeks (ADG6, FCR6, and RFI6), four and nine weeks (ADG9, FCR9, and RFI9), and four and ten weeks (ADG10, FCR10, and RFI10). To reduce the measuring bias, both the BW and FI were consistently measured using the same electronic scale (to an accuracy of 1 g). Similar to Yan et al. [30], the RFI was calculated as an individual residual (e) by regressing the ADG and average metabolic BW on the average FI (AFI): $AFI=a+b\times ADG+c\times {MBW}^{0.75}+e$. Here, a, b, and c are the regression coefficients, and MBW is the average BW during the interval of interest. In comparison with the ADG, one obvious advantage of the RFI is that it can be used to properly adjust for the individual differences in metabolic BW and ADG. For each trait, possible outliers were removed if they deviated from the mean ± 3.5 × standard deviation (SD); this threshold was empirically set.

### 2.2. Host Genotyping

On the finishing day (ten weeks of age), blood samples were individually collected from 410 broilers for genomic genotyping. The blood samples were individually collected in vacuum tubes and immediately stored at −70 °C. The genomic DNA was extracted using a Tiangen DNA Extraction Kit (Tiangen Biotech, Beijing, China) and subjected to genotyping using a chicken 55K SNP array [31] provided by Beijing Compass Biotechnology Co., Ltd. (Beijing, China). The raw SNPs were discarded if they had a calling rate < 0.9, minor allele frequency (MAF) < 0.05, or extreme deviation from the Hardy–Weinberg equilibrium (HWE, *p* < 10^−8^), determined using plink software v1.90b6.21 [32]. According to the literature, these thresholds were empirically set to maintain a balance between the number and quality of the SNPs remaining. We further imputed missing genotypes using Beagle software v5.4 and the default parameters [33]. Finally, a total of 47,872 clean SNPs, accounting for about 87% of the initially designed SNPs, remained among 409 broilers. The basic statistics of the SNPs, including the pairwise physical distance, MAF, and nucleotide diversity, were calculated using vcftools software v0.1.16 [34]. The linkage disequilibrium (LD) decay was analyzed using PopLDdecay software v3.42 [35]. Multidimensional scaling (MDS) was used for clustering the samples using plink software v1.90b6.21 [32].

### 2.3. Gut Microbiome

We collected fecal samples from the 410 broilers at six weeks of age and they were immediately snap frozen in liquid nitrogen and stored at −80 °C until further processing. The bacterial genomic DNA was extracted using a QIAamp DNA Stool Mini Kit (QIAGEN, Hilden, Germany). Subsequently, the V3-V4 region of the 16S rRNA gene was amplified using universal primers (338F—5′-ACT CCT ACG GGA GGC AGC AG-3′ and 806R—5′-GTG GAC TAC HVG GGT WTC TAA-3′) and a HOTSTAR Taq Plus Master Mix Kit (Qiagen, Shanghai, China). The PCR amplification consisted of an initial step of denaturation at 95 °C for 4 min, 20 cycles of 95 °C for 1 min, 56 °C for 45 s, and 72 °C for 1 min, and a final step of extension at 72 °C for 7 min using a Bio-Rad CFX96 thermal cycler (Bio-Rad, Hercules, CA, USA). After purification using a QIAquick PCR Purification Kit (Qiagen, Shanghai, China), Illumina sequencing libraries were constructed and sequenced by Biomarker Technologies Co., Ltd. (Beijing, China).

The sequencing data of the 16S rRNA gene were processed using Qiime2 software v2024.2 [36]. We first merged the overlapped read pairs with more than 20 nucleotides (nts), obtaining less than three mismatches using VSEARCH software v2024.2.0 [37]. The low-quality sequences were further removed if the average Q-score was lower than 30 for a sliding window of 5 nts in length. The chimeric sequences and error sequences were deduced and removed using Deblur software v2024.2.0 [38]. Finally, the first 20 nts were empirically selected and removed from the 5′ end, and all the sequences were uniquely trimmed to 400 nts. Herein, we obtained the amplicon sequence variants (ASVs), and all of them were taxonomically annotated using the q2-feature-classifier plugin v2024.2.0 in QIIME2 [39], which depends on the pre-fitted sklearn-based taxonomy classifier and Greengenes database v2022.10 [40]. We first removed the possible outliers from the samples whose library size was lower than the mean minus 3 × SD and the sparse ASVs that were present in less than 10% of the samples; these thresholds were empirically selected to achieve a balance between the number and sparsity of ASVs. The remaining ASV table, consisting of 702 ASVs and 408 samples, was randomly rarefied to the lowest library size in order to address the different sequencing depths across all the samples [41]. To calculate the microbial similarity matrix, every element of the rarefied ASV table was added by a constant of 1 and log10-transformed; each ASV was further centered and scaled. Five alpha diversity metrics of the remaining 702 ASVs, including the number of observed features, Chao1, inverse Simpson, Shannon, and Camargo’s evenness, were calculated using microbiome R package v1.23.1 [42].

### 2.4. Statistical Models

For the 13 traits examined in this study, a genomic best linear unbiased prediction (GBLUP) was performed using the linear mixed model defined as follows:

y=1μ+Wu+e,

Here, y is the vector of the individual phenotype analyzed; 1 is a vector of ones; μ is the overall mean; u is the vector of the animal additive genetic effects, together with its incidence matrix of W; and e is the vector of random residuals. Here, no known fixed effect was included, as all individuals had the same birth date, gender, feed, and raising farm. The assumed distributions of the random effects are $\mu \sim N(0,{G\sigma }_{\mu }^{2})$ and $e\sim N(0,{I\sigma }_{\mu }^{2})$, where $\sigma_{\mu }^{2}$ and $\sigma_{\mu }^{2}$ are the variances in the animal additive genetic effects and residuals, respectively. I is an identity matrix, and G is the genomic relationship matrix, which was calculated as follows [43]:

$$G=\frac{ZZ^{\prime }}{2\sum p_{i}(1-p_{i})},$$

Here, Z is the matrix of genotypes adjusted for the allele frequencies, $Z^{\prime }$ is the transpose of Z, and $p_{i}$ is the allele frequency of marker i.

By replacing the genomic data with the gut microbiome data in the GBLUP, we derived the microbial best linear unbiased prediction (MBLUP) defined as follows:

y=1μ+Xm+e,

Here, m is the vector of the random gut microbiome effect, together with its incidence matrix X. The assumed distribution of the m effect is $N(0,O\sigma_{m}^{2})$, where $\sigma_{m}^{2}$ is the variance in the random gut microbiome effect, and O is the microbial similarity matrix. O is a (co)variance matrix and calculated as $O=MM^{\prime }/(m-1)$, where M is the log-transformed and scaled count table of the ASVs, and m is the total number of ASVs [44]. The other terms are defined as above.

When combining the genomic and gut microbiome data together, the joint best linear unbiased prediction (MGBLUP) is defined as

y=1μ+Wu+Xm+e,

Here, all the terms are as defined above.

For the BW, ADG, FCR, and FRI traits at six, nine, and ten weeks of age, we calculated their genetic correlations using bivariate models:

$$\left[\begin{array}{c} y_{1}\\ y_{2} \end{array}\right]=\left[\begin{array}{cc} 1 & 0\\ 0 & 1 \end{array}\right]\left[\begin{array}{c} \mu_{1}\\ \mu_{2} \end{array}\right]+\left[\begin{array}{cc} W_{1} & 0\\ 0 & W_{2} \end{array}\right]\left[\begin{array}{c} u_{1}\\ u_{2} \end{array}\right]+\left[\begin{array}{c} e_{1}\\ e_{2} \end{array}\right],$$

Here, $y_{1}$ and $y_{2}$ are the vectors of the phenotypic records for the two traits analyzed, respectively; $\mu_{1}$ and $\mu_{2}$ are their overall means; $u_{1}$ and $u_{2}$ are the vectors of the animal additive genetic effects, together with the incidence matrices of $w_{1}$ and $w_{2}$; and $e_{1}$ and $e_{2}$ are the vectors of the random residuals. The assumed distributions of the additive genetic effects and residuals are

$$V\left[\begin{array}{c} \begin{array}{c} u_{1}\\ u_{2} \end{array}\\ e_{1}\\ e_{2} \end{array}\right]=\left[\begin{array}{cccc} {G\sigma }_{u_{1}}^{2} & G\sigma_{u_{1}u_{2}} & 0 & 0\\ G\sigma_{u_{1}u_{2}} & G\sigma_{u_{2}}^{2} & 0 & 0\\ 0 & 0 & I\sigma_{e_{1}}^{2} & I\sigma_{e_{1}e_{2}}\\ 0 & 0 & {I\sigma }_{e_{1}e_{2}} & I\sigma_{e_{2}}^{2} \end{array}\right].$$

Here, $\sigma_{u_{1}}^{2}$ and $\sigma_{u_{2}}^{2}$ are the additive genetic variances, and $\sigma_{e_{1}}^{2}$ and $\sigma_{e_{2}}^{2}$ are the residual variances for the two traits analyzed, respectively; $\sigma_{e_{1}e_{2}}$ and $\sigma_{u_{1}u_{2}}$ are the covariances between the two traits. The other terms are as defined above.

All the variance components were estimated using the average information-restricted maximum likelihood (AI-REML) method of BLUPF90 software V202301 and the default parameters [45]; the AI-REML method was used because it is computationally efficient. Accordingly, we derived the total heritability of $h^{2}=\frac{\sigma_{u}^{2}}{\sigma_{u}^{2}+\sigma_{e}^{2}}$, the total microbiability of $m^{2}=\frac{\sigma_{m}^{2}}{\sigma_{m}^{2}+\sigma_{e}^{2}}$, and the genetic correlation of $r_{g}=\frac{\sigma_{u_{1}u_{2}}}{\sqrt{\sigma_{u_{1}}^{2}\sigma_{u_{2}}^{2}}}$, which were estimated using the GBLUP, MBLUP, and MGBLUP models, respectively. Based on the joint model of MGBLUP, the direct heritability and microbiability were derived as ${\bar{h}}^{2}=\frac{\sigma_{u}^{2}}{\sigma_{u}^{2}+\sigma_{m}^{2}+\sigma_{e}^{2}}$ and ${\bar{m}}^{2}=\frac{\sigma_{m}^{2}}{\sigma_{u}^{2}+\sigma_{m}^{2}+\sigma_{e}^{2}}$, respectively. The approximated standard errors (SEs) of the estimated parameters were calculated using the Monte Carlo method [46].

## 3. Results

### 3.1. Phenotype and Correlation

The descriptive statistics of the phenotypes are shown in Table 1. The number of records range from 401 for RFI6 and RFI9 to 407 for BW9, BW10, ADG10, and FCR10, and they contain the complete phenotype, genotype, and gut microbiome data. The mean BWs measured at four, six, nine, and ten weeks of age are 735.16, 1407.78, 2509.74, and 2845.26 g, respectively. We observe the highest ADG between four and nine weeks of age (average ADG9 =50.83 g/day) and the lowest FCR between four and six weeks of age (average FCR6 = 2.12). The means (±) of RFI6, RFI9, and RFI10 are −0.28 ± 6.07, 0.19 ± 8.16, and −0.14 ± 9.31 g/day, respectively. The phenotypic correlations were estimated between all these traits (Supplementary File S1: Figure S1). BW4 had moderate-to-low positive correlations with BW6 (r = 0.79, *p* < 0.001), BW9 (r = 0.55, *p* < 0.001), and BW10 (r = 0.47, *p* < 0.001), whereas a very strong correlation (r = 0.93, *p* < 0.001) was observed between BW9 and BW10. The BW traits had moderate and strong phenotypic correlations with their corresponding FCRs (ranging from 0.59 to 0.97, *p* < 0.001), showing a decreasing trend with the FCR and RFI traits. For the two feed traits measured at the same age, only moderate phenotypic correlations (*p* < 0.001) were observed, 0.59 between FCR6 and RFI6, 0.59 between FCR9 and RFI9, and 0.57 between FCR10 and RFI10.

### 3.2. Genomic and Gut Microbiome Structures

**Figure 1:**
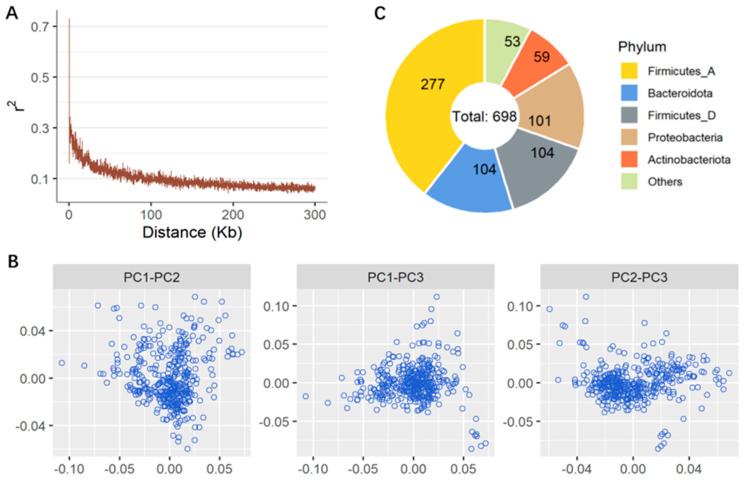
Linkage disequilibrium decay (**A**) and sample clustering (**B**) of SNPs, and taxonomical composition (**C**) of gut microbiome. r^2^ is square of correlation coefficient between allelic values at two loci. PC1, PC2, and PC3 are three top components.

All 47,872 clean SNPs were widely distributed among 32 chromosomes, and the means (±SD) were as follows: 2.6 ± 4.1 Kb for the pairwise physical distance, 0.31 ± 0.12 for the MAF, and 0.397 ± 0.109 for the nucleotide diversity. Most of the pairwise LD statistics were not higher than 0.3 (Figure 1A), and the LD obviously decayed with statistics lower than 0.1 when the physical distance was 100 Kb or greater. These statistics indicate that there was considerable genetic variation in this broiler population. Our MDS analysis based on the identity-by-state distance revealed that there was no obvious population stratification among the broilers included in this study (Figure 1B). A total of 698 out of 702 ASVs (99.4%) were taxonomically annotated at the phylum level (Figure 1C), and the most abundant phyla were Firmicutes_A (39.7%), Bacteroidota (14.9%), and Firmicutes_D (14.9%). Furthermore, 579 (82.5%) and 358 (60.0%) ASVs were successfully annotated at the genus and species levels, respectively. The relatively abundant alpha diversity was indicated by different metrics, and the means were as follows: 134.67 for the number of observed features, 164.74 for Chao1, 4.61 for inverse Simpson, 1.93 for Shannon, and 0.15 for Camargo’s evenness.

### 3.3. Heritability, Microbiability, and Genetic Correlations

The heritability and microbiability estimates are shown in Table 2. The four BW traits have comparable heritabilities, with the estimates ranging from 0.103 for BW10 to 0.156 for BW6. Three ADG traits have greater heritabilities than that of the BW traits, and the highest estimate (0.276) is similarly found at six weeks of age. Moderate and high heritabilities are found for the FCR and RFI traits, respectively, and their estimates range from 0.311 ± 0.076 for FCR9 to 0.454 ± 0.076 for FCR6, and from 0.413 ± 0.077 for RFI9 to 0.609 ± 0.076 for RFI6. The MBLUP model of BW4 does not successfully converge, whereas the successfully converged traits do not produce none-zero estimates of the microbiability. Therefore, the heritability estimates based on the MGBLUP model do not obviously differ from those based on the GBLUP model.

We further estimated the genetic correlations among the 12 traits measured at six, nine, and ten weeks of age (Table 3). BW6 only had low positive genetic correlations with BW9 and BW10, and the estimates (±SE) were 0.305 ± 0.043 and 0.443 ± 0.074, respectively. However, BW6 was strongly correlated with ADG6, with an estimate of 0.897 ± 0.343. We consistently observed negative moderate and low genetic correlations between the BW traits and the FCR and RFI traits, which means that a greater BW occurred with a lower FCR and RFI. Accordingly, strong positive correlations were observed between the FCR and RFI traits, and the estimates ranged from 0.564 ± 0.307 between FCR10 and RFI6 to 0.979 ± 0.524 between FCR9 and RFI10. For both the FCR and RFI traits, strong positive correlations, ranging from 0.739 ± 0.398 between FCR6 and FCR10 to 0.830 ± 0.268 between RFI6 and RFI9, were consistently found among the measures at the three ages of interest. However, most of these genetic correlation estimates had relatively high SEs in comparison with the means.

## 4. Discussion

The poultry industry is the most efficient system for producing high-quality animal proteins, and the consumption of poultry meat has substantially increased over the past several decades [47]. There are plenty of broiler breeds and commercial lines in China that have been extensively raised and characterized by considerable variation in growth and meat quality traits [48]. Because of their distinct genetic properties, such as the magnitudes of heritability and genetic correlations, and diverse market demands, differential breeding programs are needed across different breeds and commercial lines, for which the genetic parameters need to be specifically evaluated. Yang et al. [49] performed genomic analyses on the growth traits in a commercial nucleus breeding population of yellow-plumage chickens. Cai et al. [50] explored important candidate genes and genetic markers associated with feed efficiency and growth traits through a genome-wide association study analysis. Wen et al. [23] revealed that the gut microbiota and host genetics jointly contribute to the feed efficiency in chickens. Therefore, in this study, we investigated both the genomic and gut microbial contributions to the growth and feed efficiency traits in Dahen broilers, which have not yet been explored in this population.

The slaughter age may vary among the different breeds and lines of commercial broilers (mainly ranging from 90 to 120 days of age), with the main considerations being individual growth speed, meat quality, and market demands [51]. Furthermore, individual growth curves always differ in terms of the growth rate, adult weight, and other parameters [52]. Hence, a genetic evaluation is applied to multiple BWs measured at different ages. In this study, we evaluated the BW at four ages and found that its phenotypic correlations decreased with an the increase in the time difference; however, only low genetic correlations were observed for the BW between six and nine or ten weeks of age. Chu et al. [3] evaluated the BW of one- to six-week-old broilers raised in a commercial environment, and they found high genetic correlations between consecutive weekly BWs. These results suggest that implementing indirect selection for BW is effective only with short age intervals. When converting the BW to ADG traits, the highest ADG was found at nine weeks of age and both the phenotypic and genetic correlations of three ADGs were consistent with those of BW. Maniatis et al. [53] summarized early reports that used pedigree information to estimate the heritability of BW in broilers, and the report estimated the weight heritability from birth to 36 weeks of age to be 0.1 to 0.6. A recent study of native Thai chickens obtained similar heritability estimates for the BW and ADG at 4, 8, 12, and 16 weeks of age [54]. Therefore, in comparison with previous reports, we observed relatively low heritability estimates for the BW and ADG at different ages in Dahen broilers, which may have resulted from there being less genetic variation and/or greater environment effects in this population. The observed differences in the heritability estimates and genetic correlations may be due to differential genomic architectures across populations, for example, in terms of the allele frequencies, LD pattern, and phenotypic variations [55,56]. Furthermore, an increased environmental variance would result in lower heritability estimates [57].

In livestock and poultry, the FCR and RFI are the two most commonly used traits to measure the feed efficiency, and they have been increasingly proposed to be included in breeding programs in order to maximize productivity and minimize gross costs [58,59]. As the expected FI is obtained by regressing the ADG and average metabolic BW on the average FI, the RFI can be used to properly adjust for the growth differences among individuals, and it was first introduced in poultry in 1990 [60]. Prakash et al. [61] comprehensively reviewed the FCR and RFI in broilers in terms of their concepts and genetic parameters, and they concluded that selection based on the RFI and the FCR may be effective. Furthermore, the FCR and RFI were moderately to highly heritable, and the heritability estimates of the RFI were greater than those of the FCR. In this study, we similarly observed that the FCR and RFI were highly heritable in Dahen broilers, and that the RFI had greater heritability estimates, which are consistent with those in recent reports on broilers [62,63,64]. In this context, both the FCR and RFI could be used as efficient selection traits in breeding schemes. Furthermore, the genetic correlation between the FCR and RFI was estimated to be moderate (r = 0.77) in the broiler lines divergent for high or low abdominal fat content [63]. In this study, we also observed strong positive genetic correlations between the FCR and RFI traits, as well as between these measures at different ages. The strong or moderate genetic correlations indicate that both traits could be indirectly improved, even if only one of them is subjected to selection. Therefore, our results similarly support the notion that it is feasible to improve feed efficiency based on selection for the FCR in Dahen broilers, and the implementation of early selection may also be also possible. Genomic selection provides a promising approach to address the difficulties in the large-scale phenotypic collection of feed efficiency traits in broilers, as well as other livestock [16,17,19,65]. Therefore, we used genomic data to evaluate two feed efficiency traits, which may be used as a possible reference for implementing genomic selection in Dahen broilers.

In recent years, increasing evidence has suggested that the gut microbial community is largely involved in affecting the production and health of livestock and poultry [27,29,66,67]. Bacteroides, bifidobacteriaceae, and lactobacillaceae may potentially be used as biomarkers for feed efficiency to improve growth performance in broilers [68,69]. The intestinal microbiota exerts a positive impact on animal feed efficiency through the facilitation of nutrient absorption, the maintenance of intestinal health, and the regulation of the immune response. It was recently reported that the gut microbial community acts largely independently of the host genetics in regulating fat deposition in broilers, with an estimated microbiability of about 0.2 [70]. Yang et al. [71] investigated the dynamic changes in the gut microbial composition and suggested that they may affect the immune level, energy metabolism level, and growth in broilers. In this context, both the host genomic and gut microbiome data must be simultaneously included in the linear mixed model used for genetic evaluation, as carried out in the studies conducted by Wen et al. [23] and Jiang et al. [72] in broilers, and by Zhou et al. [25] in laying hens. These studies found that the gut microbial composition had considerable effects on various production traits. Accordingly, Wen et al. [73] discussed the emerging perspectives on the regulation of meat quality via the gut microbial community in livestock and poultry. In contrast to these previous reports, we did not observe a detectable contribution of the gut microbial community to all the traits included in this study, which may suggest that there are inter-population differences regarding the potential relationships between the gut microbiome and production traits in broilers. Certainly, we cannot absolutely exclude the possibility that the negative results observed in this study were partly caused by the statistical models used, the sequencing depth of the 16s rRNA genes, the sample size, and other factors. Therefore, we believe that comprehensive comparisons are needed in future studies. In this study, the genome and intestinal microbiome were used to analyze several important economic traits in Dahen broilers. However, additional comparisons of different statistical models, the long-read sequencing of the 16S rRNA gene, and the development of novel indicators are needed in future studies to explore potential correlations between the gut microbiota and growth and feed efficiency in broilers. Our negative results suggest that the involvement of the gut microbiota in regulating host growth and feed efficiency may vary. Therefore, some caution is needed when using gut microbiota data for the phenotypic prediction of feed efficiency and growth traits in broilers, as well as in other livestock species. Subsequently, molecular-assisted breeding could be employed to breed broiler breeds with rapid growth potential by analyzing the genes related to the growth rate in the genome. Additionally, identifying the genetic variations in feed conversion rates could help breed broiler breeds with high feed efficiency and reduce breeding costs.

## 5. Conclusions

In this study, both genomic and gut microbiome data were used to evaluate several growth and feed efficiency traits in Dahen broilers. Moderate-to-high heritabilities were found for the two feed efficiency traits, indicating that they could be genetically selected and efficiently improved. However, the gut microbial community had no detectable effect on the phenotypic variations in this study; thus, additional investigations are needed in future studies. The related parameters estimated in this study are essential in helping us to design and implement appropriate and efficient genomic selection programs in this population.

## Figures and Tables

**Table 1:** Descriptive statistics of phenotype records.

Traits ^1^	N ^2^	Mean	SD	Min	Max
BW4 (g)	405	735.16	95.22	440.00	980.00
BW6 (g)	406	1407.78	177.08	862.00	1825.00
BW9 (g)	407	2509.74	309.52	1536.00	3217.00
BW10 (g)	407	2845.26	361.56	1874.00	3749.00
ADG6 (g/day)	404	48.26	8.04	23.00	66.79
ADG9 (g/day)	406	50.83	7.47	28.06	69.03
ADG10 (g/day)	407	50.28	7.76	24.12	70.21
FCR6 (ratio)	402	2.12	0.20	1.69	2.94
FCR9 (ratio)	406	2.51	0.27	1.57	3.72
FCR10 (ratio)	407	2.71	0.35	1.91	4.93
RFI6 (g/day)	401	−0.28	6.07	−16.58	20.16
RFI9 (g/day)	401	0.19	8.16	−24.50	25.20
RFI10 (g/day)	402	−0.14	9.31	−28.91	29.97

^1^ BW4: body weight at four weeks of age; BW6: body weight at six weeks of age; BW9: body weight at nine weeks of age; BW10: body weight at ten weeks of age; ADG6: average daily gain between four and six weeks of age; ADG9: average daily gain between four and nine weeks of age; ADG10: average daily gain between four and ten weeks of age; FCR6: feed conversion ratio between four and six weeks of age; FCR9: feed conversion ratio between four and nine weeks of age; FCR10: feed conversion ratio between four and ten weeks of age; RFI6: residual feed intake between four and six weeks of age; RFI9: residual feed intake between four and nine weeks of age; RFI10: residual feed intake between four and ten weeks of age. ^2^ N: number of phenotype records; SD: standard deviation; Min: minimum; Max: maximum.

**Table 2:** Heritability and microbiability estimates (± standard error) for growth and feed efficiency traits in Dahen broilers.

Traits ^1^	Heritability ^2^		Microbiability	
	GBLUP ($h^{2}$)	MGBLUP (${\bar{h}}^{2}$)	MBLUP ($m^{2}$)	MGBLUP (${\bar{m}}^{2}$)
BW4	0.105 ± 0.080	—	—	—
BW6	0.156 ± 0.079	0.175 ± 0.082	0.021 ± 0.015	0.023 ± 0.016
BW9	0.108 ± 0.071	0.099 ± 0.071	0.002 ± 0.005	0.002 ± 0.004
BW10	0.103 ± 0.072	0.091 ± 0.071	0.002 ± 0.004	0.002 ± 0.004
ADG6	0.276 ± 0.079	0.276 ± 0.081	0.011 ± 0.012	0.013 ± 0.013
ADG9	0.187 ± 0.075	0.174 ± 0.075	0.002 ± 0.004	0.002 ± 0.004
ADG10	0.154 ± 0.074	0.140 ± 0.073	0.002 ± 0.003	0.002 ± 0.003
FCR6	0.454 ± 0.076	0.447 ± 0.076	0.002 ± 0.005	0.001 ± 0.003
FCR9	0.311 ± 0.076	0.284 ± 0.076	0.004 ± 0.006	0.003 ± 0.004
FCR10	0.396 ± 0.076	0.384 ± 0.077	0.003 ± 0.004	0.003 ± 0.005
RFI6	0.609 ± 0.076	0.604 ± 0.076	0.002 ± 0.003	0.001 ± 0.003
RFI9	0.413 ± 0.077	0.403 ± 0.077	0.006 ± 0.008	0.004 ± 0.006
RFI10	0.439 ± 0.077	0.436 ± 0.077	0.001 ± 0.002	0.000 ± 0.002

^1^ BW4: body weight at four weeks of age; BW6: body weight at six weeks of age; BW9: body weight at nine weeks of age; BW10: body weight at ten weeks of age; ADG6: average daily gain between four and six weeks of age; ADG9: average daily gain between four and nine weeks of age; ADG10: average daily gain between four and ten weeks of age; FCR6: feed conversion ratio between four and six weeks of age; FCR9: feed conversion ratio between four and nine weeks of age; FCR10: feed conversion ratio between four and ten weeks of age; RFI6: residual feed intake between four and six weeks of age; RFI9: residual feed intake between four and nine weeks of age; RFI10: residual feed intake between four and ten weeks of age. ^2^ GBLUP: genomic best linear unbiased prediction; MBLUP: microbial best linear unbiased prediction; MGBLUP: genomic and microbial joint best linear unbiased prediction; “—”: means model did not successfully converge.

**Table 3:** Estimates (lower triangular) and standard errors (upper triangular) of genetic correlations among 12 traits.

Traits ^1^	BW6 ^2^	BW9	BW10	ADG6	ADG9	ADG10	FCR6	FCR9	FCR10	RFI6	RFI9	RFI10
BW6		0.043	0.074	0.343	0.056	0.022	0.168	0.275	0.272	0.250	0.200	0.110
BW9	0.305		—	0.181	—	—	0.149	0.232	0.233	0.016	0.052	0.245
BW10	0.443	—		0.206	—	0.293	0.201	0.343	0.163	0.021	0.092	0.219
ADG6	0.897	0.582	0.595		0.085	0.136	0.020	0.150	0.104	0.116	0.145	0.014
ADG9	0.193	—	—	0.415		—	0.137	0.226	0.312	0.007	0.002	0.199
ADG10	0.225	—	0.981	0.450	—		0.272	0.434	0.266	0.010	0.147	0.284
FCR6	−0.257	−0.366	−0.476	−0.147	−0.299	−0.582		0.445	0.398	0.454	0.441	0.458
FCR9	−0.475	−0.597	−0.562	0.206	−0.547	−0.935	0.826		0.420	0.395	0.377	0.524
FCR10	−0.490	−0.576	−0.435	0.152	−0.652	−0.625	0.739	0.813		0.307	0.533	0.324
RFI6	−0.487	−0.029	−0.038	0.220	−0.004	−0.180	0.885	0.732	0.564		0.268	0.529
RFI9	−0.405	−0.065	−0.185	0.312	0.035	−0.247	0.793	0.749	0.853	0.830		0.359
RFI10	−0.237	−0.400	−0.464	0.022	−0.324	−0.509	0.837	0.979	0.954	0.736	0.791	

^1^ BW4: body weight at four weeks of age; BW6: body weight at six weeks of age; BW9: body weight at nine weeks of age; BW10: body weight at ten weeks of age; ADG6: average daily gain between four and six weeks of age; ADG9: average daily gain between four and nine weeks of age; ADG10: average daily gain between four and ten weeks of age; FCR6: feed conversion ratio between four and six weeks of age; FCR9: feed conversion ratio between four and nine weeks of age; FCR10: feed conversion ratio between four and ten weeks of age; RFI6: residual feed intake between four and six weeks of age; RFI9: residual feed intake between four and nine weeks of age; RFI10: residual feed intake between four and ten weeks of age. ^2^ “—”: did not converge.

## Data Availability

Data are available upon request from the corresponding author.

## Supplementary Materials

The following supporting information can be downloaded at: https://www.mdpi.com/article/10.3390/ani14243615/s1, Figure S1: Phenotypic correlations among all traits analyzed in this study; Table S1: Feed composition and nutritional values used in this study.
