# Supplementary material for: Genomic and Gut Microbiome Evaluations of Growth and Feed Efficiency Traits in Broilers
Source: Animals (Basel). 2024 Dec 15;14(24):3615. doi: 10.3390/ani14243615 (PMC11672845; doi:10.3390/ani14243615)
Supplement: Supplementary file 1 [file animals-14-03615-s001.zip › Supplementary Figure.pdf]

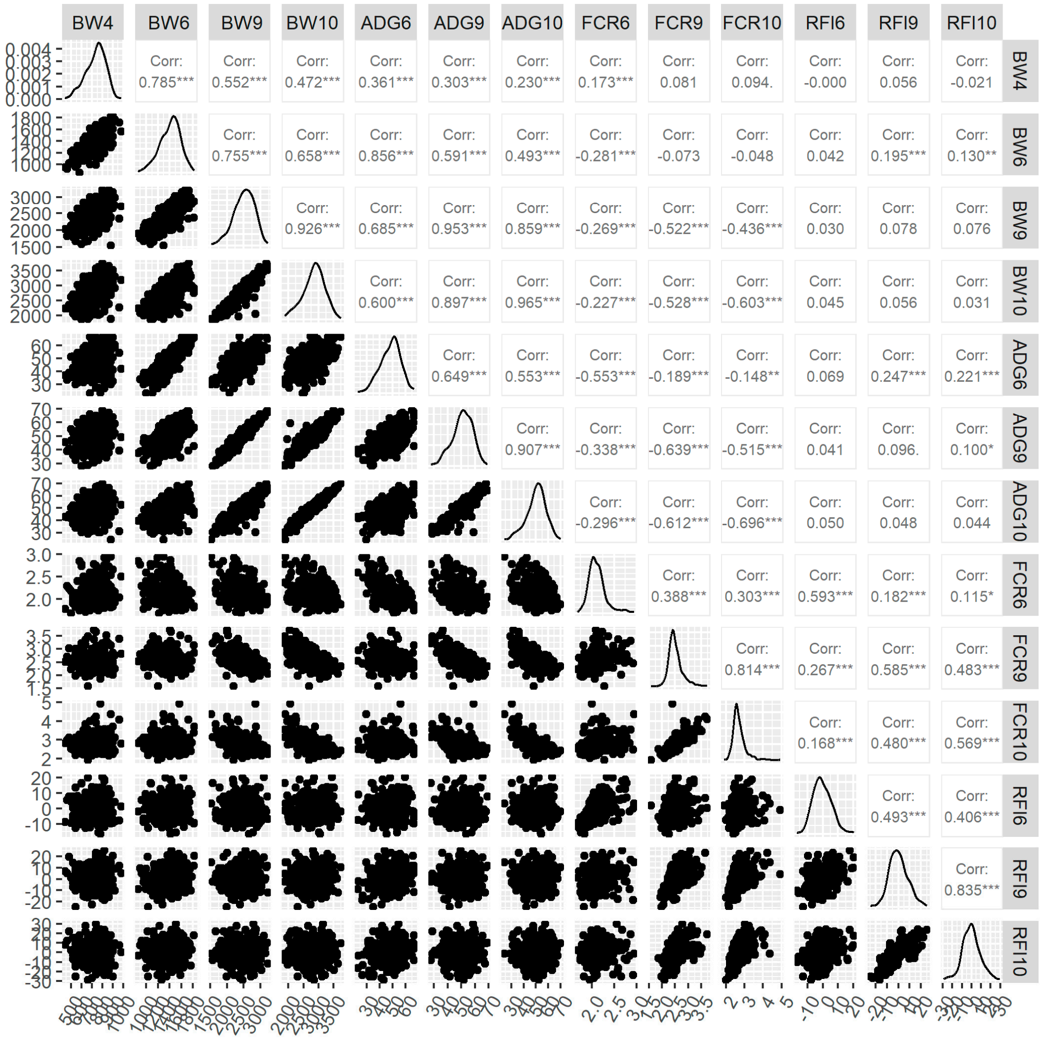

Figure S1. Phenotypic correlations among all traits analyzed in this study. BW4: body weight at four weeks of age; BW6: body weight at six weeks of age; BW9: body weight at nine weeks of age; BW10: body weight at ten weeks of age; ADG6: average daily gain between four and six weeks of age; ADG9: average daily gain between four and nine weeks of age; ADG10: average daily gain between four and ten weeks of age; FCR6: feed conversion ratio between four and six weeks of age; FCR9: feed conversion ratio between four and nine weeks of age; FCR10: feed conversion ratio between four and ten weeks of age; RFI6: residual feed intake between four and six weeks of age; RFI9: residual feed intake between four and nine weeks of age; RFI10: residual feed intake between four and ten weeks of age.
