# Supplementary material for: Genomic and Gut Microbiome Evaluations of Growth and Feed Efficiency Traits in Broilers
Source: Animals (Basel). 2024 Dec 15;14(24):3615. doi: 10.3390/ani14243615 (PMC11672845; doi:10.3390/ani14243615)
Supplement: Supplementary file 1 [file animals-14-03615-s001.zip › Supplementary Table.pdf]

Table S1. Feed composition and nutritional values used in this study.

Items	1 to 21 days of age	22 to 42 days of age	43 to 70 days of age
Ingredients			
Corn	61.3	66.7	69.5
Soybean meal	26.0	20.4	17.0
Corn gluten meal	4.0	4.0	4.0
Distiller Dried Grains with Solubles	3.0	3.0	3.0
Soybean oil	1.0	1.5	2.6
Limestone	1.4	1.4	1.3
CaHPO4	1.5	1.3	1.0
NaCl	0.3	0.3	0.3
L-Lysine HCl	0.38	0.33	0.3
DL-Methionine	0.20	0.18	0.12
L-Threonine	0.06	0.07	0.07
Choline chloride	0.08	0.08	0.08
Vitamin and minerals premix	0.781	0.742	0.733
Total	100	100	100
Nutrient levels[4]			
Metabolizable energy (MJ/kg)	12.14	12.45	12.91
Crude protein	19.91	17.63	16.11
Calcium	0.96	0.87	0.76
Total phosphorus	0.60	0.54	0.46
Lysine	1.12	1.03	0.91
Methionine	0.48	0.45	0.42

[1]The vitamin and minerals premix provided the following per kg of diets: VA 9000 IU, VD3 1800 IU, VE 35 IU, VK3 3.1 mg, VB1 2.8 mg, VB2 6.7 mg, VB6 4.5 mg, VB12 31 μg, biotin 0.24 mg, pantothenic acid 15.8 mg, folic acid 1.1 mg, nicotinic acid 45 mg, Fe 140 mg, Cu 20 mg, Mn 125 mg, Zn 110 mg, I 0.40 mg, Se 0.50 mg.

[2]The vitamin and minerals premix provided the following per kg of diets: VA 8800 IU, VD3 1700 IU, VE 35 IU, VK3 2.9 mg, VB1 2.5 mg, VB2 6.3 mg, VB6 4.2 mg, VB12 29 μg, biotin 0.20 mg, pantothenic acid 14.5 mg, folic acid 1.02 mg, nicotinic acid 42 mg, Fe 125 mg, Cu 17 mg, Mn 105 mg, Zn 110 mg, I 0.50 mg, Se 0.5 mg.

[3]The vitamin and minerals premix provided the following per kg of diets: VA 8600 IU, VD3 1600 IU, VE 34 IU, VK3 2.7 mg, VB1 2.4 mg, VB2 6.0 mg, VB6 4.0 mg, VB12 28 μg, biotin 0.2 mg, pantothenic acid 14 mg, folic acid 1.0 mg, nicotinic acid 40 mg, Fe 125 mg, Cu 17 mg, Mn 105mg, Zn 110 mg, I 0.50 mg, Se 0.5 mg.

[4]Metabolizable energy was a calculated value, while the others were measured values.
